# Repeat crossclamp after failed initial degenerative mitral valve repair is safe and successful

**DOI:** 10.1016/j.xjon.2023.08.021

**Published:** 2023-09-16

**Authors:** Catherine M. Wagner, Whitney W. Fu, Alexander A. Brescia, Robert B. Hawkins, Matthew A. Romano, Gorav Ailawadi, Steven F. Bolling

**Affiliations:** a Department of Cardiac Surgery, University of Michigan Medicine, Ann Arbor, Mich; b Department of General Surgery, University of Michigan Medicine, Ann Arbor, Mich

**Keywords:** degenerative mitral valve disease, mitral valve repair

## Abstract

**Objective:**

Surgical risk and long-term outcomes when re-crossclamp is required during degenerative mitral valve repair are unknown. We examined the outcomes of patients who required re-crossclamp for mitral valve reintervention.

**Methods:**

Adults undergoing mitral valve repair for degenerative mitral valve disease at a single center from 2007 to 2021 who required more than 1 crossclamp for mitral valve reintervention were included. Outcomes including major morbidity and 30-day mortality were collected. Kaplan–Meier analysis characterized survival and freedom from recurrent mitral regurgitation.

**Results:**

A total of 69 patients required re-crossclamp for mitral valve reintervention. Of those, 72% (n = 50) underwent successful re-repair and the remaining underwent mitral valve replacement (28%, n = 19). Major morbidity occurred in 23% (n = 16). There was no 30-day mortality, and median long-term survival was 10.9 years for those undergoing re-repair and 7.2 years for those undergoing replacement (*P* = .79). Midterm echocardiography follow-up was available for 67% (33/50) of patients who were successfully re-repaired with a median follow-up of 20 (interquartile range, 7-37) months. At late follow-up, 90% of patients had mild or less mitral regurgitation. Of those re-repaired, 2 patients later required mitral valve reintervention.

**Conclusions:**

Patients requiring re-crossclamp for residual mitral regurgitation had low perioperative morbidity and no mortality. Most patients underwent successful re-repair (vs mitral valve replacement) with excellent valve function and long-term survival. In the event of unsatisfactory repair at the time of mitral valve repair, attempt at re-repair is safe and successful with the appropriate valvar anatomy.

## Graphical Abstract

**Figure:**
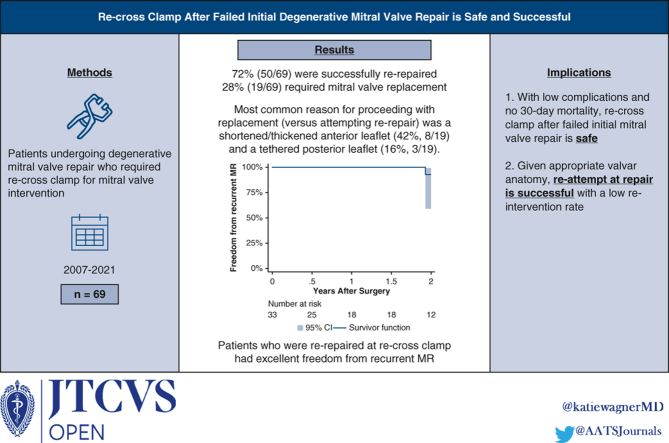

**Figure:**
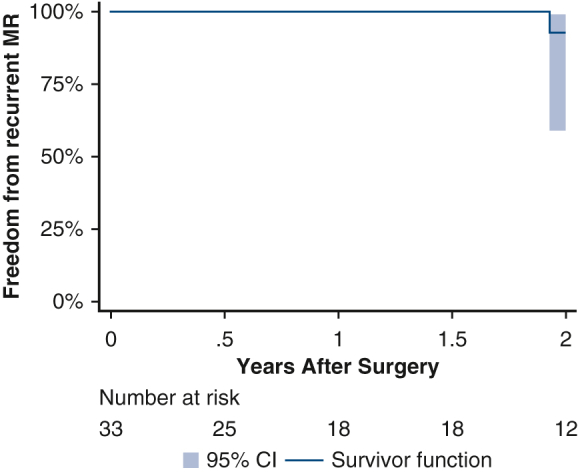
There is excellent freedom from recurrent MR after re-repair.

**Central Message**

Given appropriate anatomy, reattempt at mitral valve repair is safe for patients who require re-crossclamp for mitral valve reintervention.

**Perspective**

If re-crossclamp is required for residual MR or SAM, reattempt at mitral valve repair is safe and feasible. Rather than proceeding directly to MVR upon re-crossclamp, mitral valve re-repair should be attempted given appropriate valvar anatomy.

See Discussion on Page 218.

Mitral valve repair for degenerative mitral valve disease is the gold standard treatment and has been shown to lead to improved survival versus mitral valve replacement (MVR).^1^, ^2^, ^3^, ^4^ However, mitral valve repair is a technically challenging operation, and attempts at repair may not always be successful.^5^ After crossclamp removal, residual mitral regurgitation (MR) or systolic anterior motion (SAM) of the mitral valve suggests an inadequate initial attempt at mitral valve repair. Residual MR and SAM are unsatisfactory repair results; thus, the surgeon should consider re-crossclamping to reintervene on the mitral valve.

However, outcomes of patients with degenerative mitral valve disease requiring re-crossclamp are not well described.^5^ In addition, upon re-crossclamp, it is not known if a repeat attempt at mitral valve repair is safe or successful. It is possible that surgeons just proceed to MVR on re-crossclamp,^6^^,^^7^ but this may not be necessary.

In this study, we examined patients with degenerative mitral valve disease undergoing scheduled mitral valve repair who required re-crossclamp for mitral valve reintervention. Specifically, we sought to describe the postoperative morbidity and mortality of this population, and to compare long-term outcomes of patients who underwent mitral valve re-repair versus MVR.

## Patients and Methods

### Data Source

This study was deemed exempt from review by the University of Michigan Institutional Review Board (HUM00148119, January 23, 2023). Patient demographics, operative characteristics, and outcomes data were collected through the University of Michigan institutional component of the Society of Thoracic Surgeons Adult Cardiac Surgery Database. Intraoperative decision-making (ie, decision to proceed with MVR), follow-up echocardiogram data, and mitral valve reintervention data were assessed through chart review.

### Patient Population

**Figure E1:**
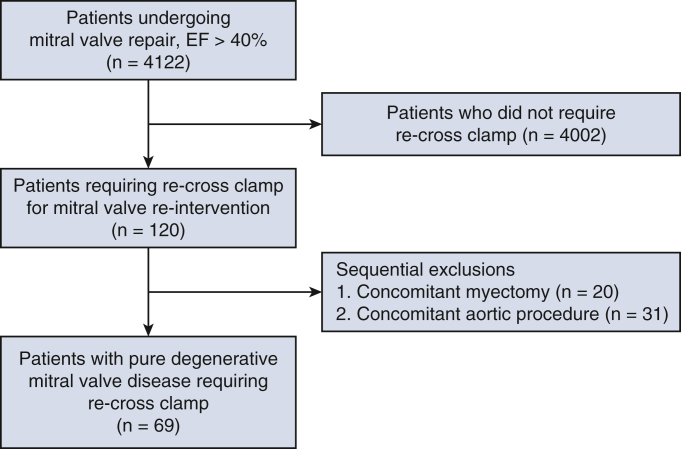
CONSORT diagram.

Adult patients with an ejection fraction more than 40% who were scheduled for mitral valve repair for degenerative mitral valve repair from 2007 to 2021 at our institution were included (n = 4130). Patients with ejection fraction less than 40% were excluded to ensure a pure degenerative mitral valve disease population was isolated. Patients who did not require re-crossclamp were excluded (4002). Patients were excluded if they underwent concomitant myectomy (n = 20) or concomitant aortic procedure (n = 31) (Figure E1).

### Operative Technique

All patients first underwent attempted mitral valve repair for degenerative mitral valve disease. For mode of thorax entry, sternotomy and right thoracotomy are both used for isolated mitral surgery, and the preferred approach is largely surgeon dependent. The technique to perform mitral valve repair for degenerative valve disease varies by surgeon and patient anatomy, but includes resection of redundant leaflet tissue, chord placement, and annuloplasty. First, competency of the mitral valve repair is assessed with the crossclamp on with a bulb syringe. When that appears satisfactory, the atriotomy is closed and the crossclamp is removed. The mitral valve repair is then fully assessed by transesophageal echocardiography. If the repair has mild or less MR by transesophageal echocardiography, the patient is decannulated from cardiopulmonary bypass and the operation concludes. If the repair is unsatisfactory due to residual MR (moderate or more per the transesophageal echocardiogram), the crossclamp is reapplied for mitral valve reintervention. If the repair is unsatisfactory due to SAM, medical management is first used. If the SAM persists despite medical management and adequate left ventricular filling, the crossclamp is reapplied for mitral valve reintervention.

Strategies for re-repair if the crossclamp is reapplied for residual MR vary depending on the etiology of the recurrent MR. Strategies for re-repair if the crossclamp is reapplied for SAM focus on reducing the height of the posterior leaflet. This can be accomplished by enlarging the annuloplasty ring, resecting more leaflet, or possibly performing an edge-to-edge repair.

### Intraoperative Assessment of the Valve and Decision Making

Operative reports were reviewed to determine whether patients required re-crossclamp for mitral valve reintervention due to residual MR or SAM. Surgeon decision-making in electing to proceed with MVR (vs continuing to attempt re-repair) was identified in the operative report.

### Outcomes

Primary outcomes were major morbidity and short- and long-term mortality. Major morbidity was defined in accordance with the Society of Thoracic Surgeons Performance Measures and includes having any of the following postoperative complications: (1) reoperations for any cardiac reason, including valvular dysfunction or postoperative bleeding; (2) renal failure; (3) deep sternal wound infection; (4) prolonged ventilation/intubation; and (5) cerebrovascular accident/permanent stroke. The 30-day mortality was defined as in-hospital or within 30 days of the index operation. Date of follow-up was defined as the most recent clinic or chart encounter in the electronic medical record. Date of death was assessed through retrospective chart review.

Secondary outcomes examined the postoperative echocardiogram outcomes and need for mitral valve reintervention. Data were collected using the most recent echocardiogram available. Atrioventricular valve regurgitation grade was coded 0 for trivial/none, 1 for mild, 2 for moderate, 3 for moderate-severe, and 4 for severe. Recurrent MR was defined as grade 2 or greater MR. Need for mitral valve reintervention was assessed with chart review.

### Statistical Analysis

**Figure E2:**
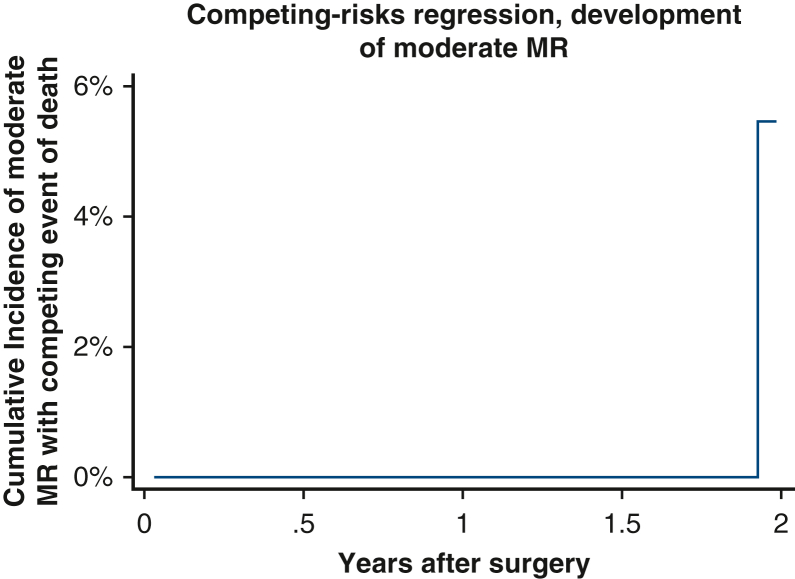
Fine-Gray model of for development of moderate MR with death as a competing event. *MR*, Mitral regurgitation.

Descriptive data were collected and analyzed using frequencies and proportions. Categorical variables are presented as percentages of the total number of patients. Continuous variables are presented as median with interquartile range (IQR). Comparisons between groups were performed using Wilcoxon rank-sum for continuous variables and chi-square for categorical variables. Some data were missing for long-term echocardiographic follow-up, and the follow-up data presented outcomes of patients with long-term follow-up data available. Kaplan–Meier analysis was performed to characterize survival. A Fine-Gray model was performed to calculate the development of recurrent MR with the competing risk of death. No patients who underwent mitral valve repair died; thus, the Fine-Gray and Kaplan–Meier time to event analysis for development of MR among patients who underwent mitral valve repair were identical. The cumulative incidence function is shown in Figure E2, and occurrence of each event and 95% CIs are shown in Table E1. To graph freedom from recurrent MR, Kaplan–Meier analysis was performed. Analyses were performed using Stata 17.0 (StataCorp LLC).

## Results

### Patient Characteristics

A total of 69 patients underwent reintervention on the mitral valve after failed initial mitral valve repair, of whom 50 were successfully re-repaired and 19 required MVR. The median age was 62 years (IQR, 52-69), and 39% (n = 14) were female. The median ejection fraction was 65% (IQR, 60-70), and 9% (n = 6) received redo cardiac surgery. Most cases were elective, although 6% (n = 4) had an urgent procedure. Demographics did not differ between patients who underwent re-repair versus eventual replacement (Table 1).

**Table 1:** Comparison of demographics of patients requiring re-crossclamp for mitral valve reintervention who underwent mitral valve replacement versus re-repair

Variable	Total (n = 69)	Required MVR (n = 19)	Mitral valve re-repair (n = 50)	*P* value
Age, y (median, IQR)	62 (52-69)	64 (55-69)	62 (48-69)	.536
Female	27 (39%)	9 (47%)	18 (36%)	.387
Diabetes	14 (20%)	4 (21%)	10 (20%)	.923
Hypertension	45 (65%)	13 (68%)	32 (64%)	.731
Preoperative atrial fibrillation	16 (23%)	4 (21%)	12 (24%)	.796
Preoperative ejection fraction	65 (60-70)	65 (53-70)	65 (60-66)	.907
Redo surgery	6 (9%)	1 (5%)	5 (10%)	.533
Urgent	4 (6%)	0 (0%)	4 (8%)	.204

*MVR*, Mitral valve replacement; *IQR*, interquartile range.

### Operative Characteristics

Concomitant procedures included tricuspid valve procedures (33%, n = 23), aortic valve replacement (12%, n = 8), and atrial fibrillation procedures (22%, n = 15). Most patients required 2 crossclamps (78%, 54), followed by 3 crossclamps (20%, 14), and 1 patient required 4 crossclamps (2%, 1). Although patients who required 3 crossclamps were more likely to undergo MVR, the 1 patient who required 4 crossclamps was successfully re-repaired (Table 2). The median crossclamp time for the entire cohort was 148 (IQR, 107-209) minutes, and the median cardiopulmonary bypass time was 210 (IQR, 147-277) minutes. These were both longer in the MVR versus re-repair group, likely because those who required MVR were more likely to require 3 crossclamps than the re-repair group.

**Table 2:** Operative characteristics of patients requiring re-crossclamp for reintervention of the mitral valve

Variable	Total (n = 69)	Required MVR (n = 19)	Mitral valve re-repair (n = 50)	*P* value
No. of crossclamps				.019
2	54 (78%)	11 (58%)	43 (86%)	
3	14 (20%)	8 (42%)	6 (12%)	
4	1 (2%)	0 (0%)	1 (2%)	
Crossclamp time (min) (median, IQR)	148 (107-209)	173 (138-238)	143 (103-186)	.028
Cardiopulmonary bypass time (min) (median, IQR)	210 (147-277)	231 (189-314)	194 (141-251)	.019
Reason for re-crossclamp				.019
SAM (vs recurrent MR)	12 (17%)	0/12 (0%)	12/12 (100%)	
Recurrent MR	57 (83%)	19/57 (33%)	38/57 (67%)	
Concomitant procedures				
Tricuspid valve procedure	23 (33%)	7 (37%)	16 (32%)	.703
Aortic valve procedure	8 (12%)	0 (0%)	8 (16%)	.064
Atrial fibrillation procedure	15 (22%)	3 (16%)	12 (24%)	.460

*MVR*, Mitral valve replacement; *IQR*, interquartile range; *SAM*, systolic anterior motion; *MR*, mitral regurgitation.

**Figure 1:**
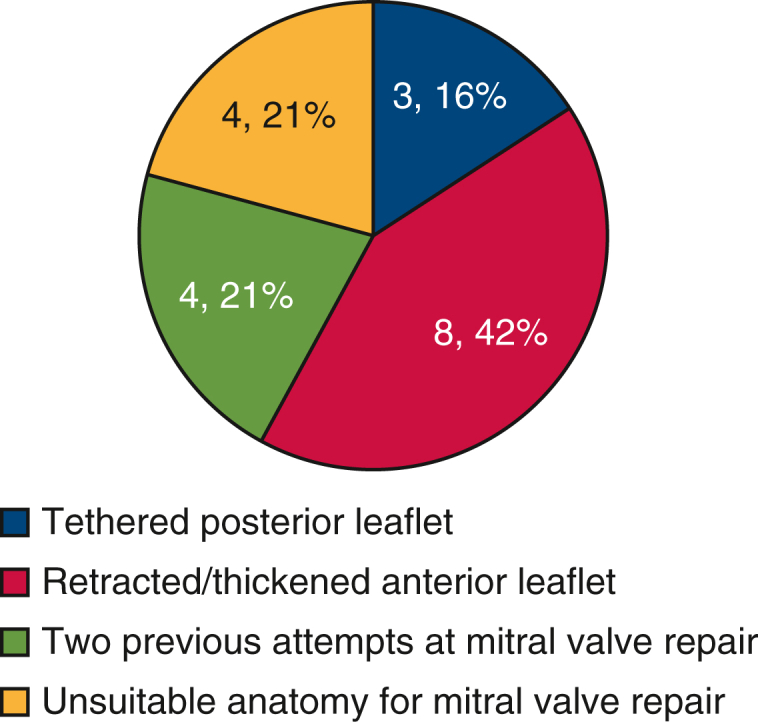
Reason for proceeding with MVR.

Of the 12 patients who had repeat crossclamp due to SAM, all were successfully re-repaired. Reasons for proceeding with MVR included a shortened/thickened anterior leaflet (42%, 8/19), a determination that the anatomy was not amenable to repair (21%, 4/19), 2 failed attempts at MVR requiring a third crossclamp (21%, 4/19), and a tethered posterior leaflet (16%, 3/19) (Figure 1).

### Outcomes

The median intensive care unit length of stay was 3 (IQR, 1-5) days and did not differ between patients who underwent re-repair versus replacement (Table 3). Major morbidity occurred in 20% (10/50) of patients re-repaired and 32% (6/19) of patients who required MVR (*P* = .309). The most common complication was prolonged ventilation, followed by the need for reoperation, renal failure, postoperative stroke, and sternal wound infection. No patients were an operative mortality. The median long-term follow up was 12 months (IQR, 3-47 months). The median 5-year survival was 93% (95% CI, 60-99) for patients who underwent MVR and 96% (95% CI, 73-99) for those who were re-repaired (*P* = .79).

**Table 3:** Outcomes of patients requiring re-crossclamp for mitral valve reintervention

Variable	Total (n = 69)	Required MVR (n = 19)	Mitral valve re-repair (n = 50)	*P* value
ICU length of stay, d	3 (1-5)	4 (1-6)	3 (1-5)	.472
ICU readmission	3 (4%)	1 (5%)	2 (4%)	.818
Major morbidity	16 (23%)	6 (32%)	10 (20%)	.309
Sternal wound infection	1 (1%)	0 (0%)	1 (2%)	.535
Postoperative stroke	1 (1%)	0 (0%)	1 (2%)	.535
Prolonged ventilation	12 (17%)	5 (26%)	7 (14%)	.228
Postoperative renal failure	2 (3%)	0 (0%)	2 (4%)	.376
Postoperative reoperation	4 (6%)	2 (11%)	2 (4%)	.300
Operative mortality	0% (0)	0% (0)	0% (0)	-
Total length of stay, d (median, IQR)	7 (5-10)	10 (8-21)	6.5 (5-8)	<.001
Discharge to subacute rehab or nursing facility	6 (9%)	2 (11%)	4 (8%)	.739
30-d readmission	8 (12%)	1 (5%)	7 (14%)	.311

*MVR*, Mitral valve replacement; *ICU*, intensive care unit; *IQR*, interquartile range.

Patients who were re-repaired had a shorter length of stay than those who required MVR: 6.5 days (IQR, 5-8) versus 10 days (IQR, 8-21; *P* < .001). Most patients were discharged home (vs subacute rehabilitation facility or nursing facility) (91%, 60/69), and there was no difference in discharge location between patients who underwent re-repair versus MVR.

**Figure 2:**
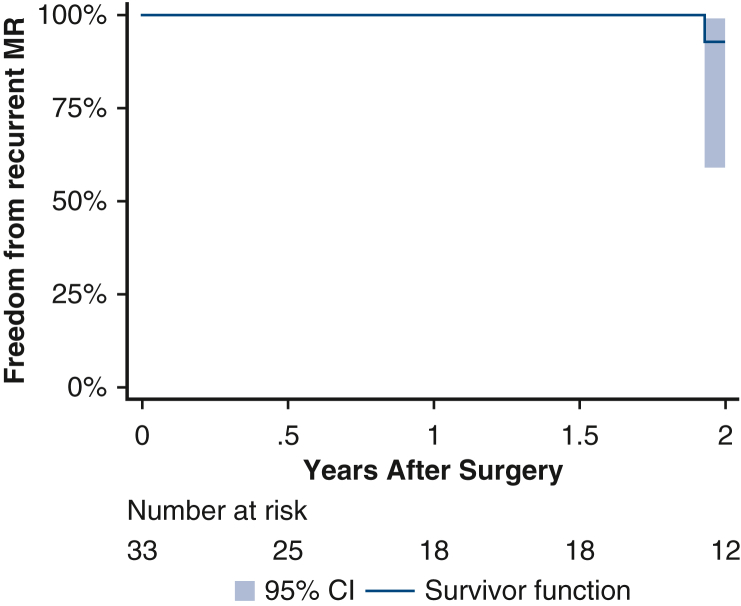
Freedom from recurrent MR among patients who underwent mitral valve repair at re-crossclamp. *MR*, Mitral regurgitation.

Among patients who were re-repaired, 66% (33/50) had a follow-up echocardiogram at a median follow up of 20 (IQR, 7-37) months. Freedom from recurrent MR at 2 years was 95% (95% CI, 0.3-18) (Figure 2). Two patients required further reintervention, both for recurrent SAM. The first patient's index operation required re-crossclamp for SAM. Postoperatively, the patient had orthostatic hypotension and symptoms consistent with left ventricular outflow tract obstruction, and repeat echocardiogram showed SAM. He underwent reoperation 13 days after his index operation with a successful mitral valve repair and was discharged 4 days later. The second patient's index operation required re-crossclamp for recurrent MR. Her postoperative course was uneventful, and she was discharged home. However, 5 weeks after discharge, she noted increased dyspnea on exertion. A follow-up echocardiogram showed SAM. This was initially treated with beta-blockade and hydration; however, symptoms persisted, and 75 days after her first operation she underwent successful re-repair of her mitral valve.

## Discussion

Our study evaluating patients with degenerative mitral valve disease who required repeat crossclamp for mitral valve reintervention has 2 principal findings. First, patients who required repeat crossclamp had low operative morbidity and no operative mortality. Second, patients who were successfully re-repaired (vs underwent MVR) had excellent repair durability and midterm outcomes. Taken together, reattempt at mitral valve repair is safe for patients who require re-crossclamp for mitral valve reintervention.

Although crossclamp times were prolonged, patients still had low rates of complications and no operative mortality. Prolonged crossclamp time is often associated with increased morbidity and mortality.^8^ However, prior work examined the association between aortic crossclamp time and morbidity and mortality in patients undergoing mitral valve repair, and similarly found that a longer aortic crossclamp time was not associated with higher morbidity and mortality.^9^ Many patients undergoing mitral valve repair for degenerative disease tend to be younger with fewer comorbidities than patients undergoing other forms of cardiac surgery.^4^ These younger and healthier patients may be able to withstand a longer crossclamp time with fewer complications. Ultimately, the surgeon understands and must consider all clinical factors for each specific patient. However, if the initial mitral valve repair appears unsatisfactory due to residual MR or SAM on transesophageal echocardiogram, our data suggest that it is safe to reapply the aortic crossclamp and reintervene on the mitral valve.

After reapplying the crossclamp, surgeons must reconsider if the patient has suitable anatomy to reattempt mitral valve repair.^10^, ^11^, ^12^, ^13^, ^14^, ^15^, ^16^, ^17^ Oftentimes, such a determination may be made based on the transesophageal echocardiogram. Anatomic factors that may influence this include adequacy of leaflet tissue to reattempt repair or if the posterior leaflet is restricted/tethered.^13^ Not every valve will be able to be re-repaired, and in this series just over one-quarter required MVR. However, for those with amenable anatomy, at our center we use 3-dimensional transesophageal echocardiography to aid in visualizing the mitral valve to guide re-repair strategy. Within this series, 72% of patients (50/69) were successfully re-repaired, and all patients with SAM were re-repaired. Of note, and echoing prior studies, patients who were re-repaired had excellent midterm survival and freedom from recurrent MR.^10^^,^^12^^,^^17^ Our data suggest that given suitable anatomy, re-repair is feasible and leads to excellent midterm outcomes.

Upon re-crossclamp, some surgeons may be tempted to immediately proceed with MVR instead of reattempting to repair.^6^^,^^7^ This may be due to concerns of failure of attempted re-repair with resultant prolonged crossclamp time.^8^ However, surgeons must balance the importance of a short crossclamp time with the goal of providing the best possible surgical mitral valve intervention for their patient. In patients with degenerative mitral valve disease, MVR leads to worse long-term survival than mitral valve repair.^1^, ^2^, ^3^, ^4^^,^^18^ If the patient has suitable anatomy, re-repair should be attempted to give that patient the best possible long-term outcome.

### Study Limitations

Our study has several limitations. First, the need for re-crossclamp during degenerative mitral valve repair was rare, and thus we have a small sample size. The small sample size increases the risk of a type 1 error and may limit reproducibility. However, this is among the largest studies to examine the outcomes of re-crossclamp for mitral valve repair at a high-volume center^19^ and can still provide insight into management practices for these patients. Next, there was missingness in our follow-up echocardiograms, which may introduce bias, and the median time to echocardiogram was only 1 year, lacking long-term durability data. However, most patients who have recurrent MR due to technical failure of a mitral valve repair have recurrence within the first 2 years postoperatively. Our data provide value in the midterm durability of mitral valve repair after re-crossclamp. Finally, this study occurred at a Mitral Foundation/American Heart Association Reference center, which may limit generalizability. However, the importance of experienced centers performing mitral valve repair has been increasingly recognized to optimize outcomes and increase likelihood of successful mitral valve repair (vs MVR).^20^

## Conclusions

**Figure 3:**
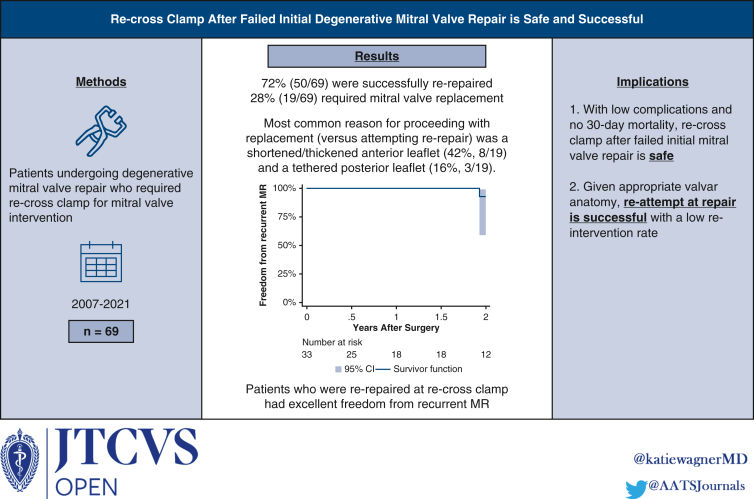
Graphical abstract. *MR*, Mitral regurgitation.

Patients requiring re-crossclamp for initial failed mitral valve repair had low morbidity and no operative mortality (Figure 3). A high percentage of patients underwent successful re-repair versus replacement. Given the appropriate anatomy, mitral valve re-repair is safe and effective and should be attempted instead of proceeding directly to MVR.

### Webcast

You can watch a Webcast of this AATS meeting presentation by going to: https://www.aats.org/resources/repeat-cross-clamp-for-residual-mitral-regurgitation-during-degenerative-mitral-repair-is-safe.

**Figure:**
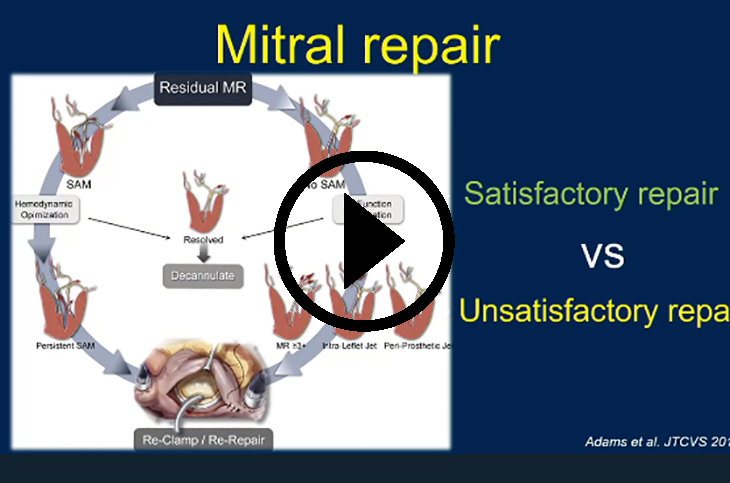

## Conflict of Interest Statement

G.A. is a consultant for Abbott, Edwards, Medtronic, Anteris, Atricure, and Gore. All other authors reported no conflicts of interest.

The *Journal* policy requires editors and reviewers to disclose conflicts of interest and to decline handling or reviewing manuscripts for which they may have a conflict of interest. The editors and reviewers of this article have no conflicts of interest.

## Figures and Tables

**Table E1:** Development of moderate mitral regurgitation with death as a competing event

Time point	Cumulative incidence function (95% CI)
0.5 y	0%
1 y	0%
1.5 y	0%
2 y	4.5% (0.3-18.2)
